# Epidemiology of distal radius fracture in Akershus, Norway, in 2010–2011

**DOI:** 10.1186/s13018-018-0904-0

**Published:** 2018-08-13

**Authors:** Håkon With Solvang, Robin Andre Nordheggen, Ståle Clementsen, Ola-Lars Hammer, Per-Henrik Randsborg

**Affiliations:** 1 The Department of Orthopedic Surgery, Akershus University Hospital, 1478 Lørenskog, Norway; 2 The Faculty of Medicine, The University of Oslo, Oslo, Norway

**Keywords:** Distal radius fracture, Epidemiology, Volar locking plate, AO classification

## Abstract

**Background:**

Several studies published over the last decade indicate an increased incidence of distal radius fractures (DRF). With Norway having one of the highest reported incidence of DRFs, we conducted a study to assess the epidemiology of DRFs and its treatment in the catchment area of Akershus University Hospital (AHUS).

**Methods:**

Patients 16 years or older who presented to AHUS with an acute DRF during the years 2010 and 2011 were prospectively recorded and classified according to the AO fracture classification system. The mechanism of injury and treatment modality were noted.

**Results:**

Overall, 1565 patients with an acute DRF presented to the institution in 2010–2011, of which 1134 (72%) were women. The overall annual incidence was 19.7 per 10,000 inhabitants 16 years or older. Women had an exponential increase in incidence after the age of 50, though the incidence for both genders peaked after the age of 80 years. There was an even distribution between extra- and intra-articular fractures. Falling while walking outside was the most common mechanism of injury. Of the 1565 registered, 418 (26.7%) patients underwent surgery, with a volar locking plate being the preferred surgical option in 77% of the cases.

**Conclusion:**

The overall incidence of distal radius fractures was lower in our study than earlier reports from Norway. Postmenopausal women had a higher risk of fracture than the other groups, and low-energy injuries were most dominant. 26.7% were treated operatively, which is higher than earlier reports, and might reflect an increasing preference for surgical treatment.

## Background

Distal radius fracture (DRF) is one of the most common fractures in the human body. It has a bimodal distribution, with a peak incidence among young patients with high-energy traumas and elderly patients with low-energy falls [1–4]. In addition to the individual morbidity, considerable financial resources are spent investigating and treating this injury [5]. Absence from work adds to this socioeconomic load. From the 1950s, a continuous increase in the incidence of DRFs has been described. However, some recent studies describe a stabilizing trend [2, 6], or even a decline in incidence [1, 7], especially in young postmenopausal women [2, 4, 8].

Treatment of DRFs varies, not only between countries but also between national regions and individual hospitals. Following the introduction of volar locking plates (VLP) in the early 2000s, surgical management seems to increase at the cost of conservative treatment [7].

The purpose of the current study is to map the incidence of DRFs among the adult population in the catchment area of Akershus University Hospital (AHUS). Moreover, we studied the distribution between genders, the mechanism of injury, the type of fracture, and the choice of treatment.

## Methods

AHUS is a level 2-university hospital covering suburban and urban regions in and outside Oslo, Norway. Patients 16 years or older who presented to AHUS with an acute DRF between January 1, 2010, and December 31, 2011, were eligible for inclusion. A DRF was defined as a fracture of the radius within 3 cm from the radiocarpal joint [9]. To be included in the study, patients had to live within the catchment area of AHUS. Patients primarily treated in another hospital but who had their follow-up appointments at AHUS were also included in this study. All data were registered prospectively in a database. Patient demographics (gender and age), the mechanism of injury, and time of injury were registered. The fractures were classified according to the *Arbeitsgemeinschaft fur Osteosyntesefragen* (AO) fracture classification system by a senior orthopedic consultant, to either a type A (extra-articular), type B (partial articular), or type C (complete intra-articular) fracture. To verify the total number of patients and type of treatment, a search was made in the hospital’s electronic files by entering the diagnosis code. The medical records and the radiographs for each registered patient in the prospective database were then retrospectively reviewed and verified from the electronic files.

The age and gender distribution in the catchment area of AHUS were available from Statistics Norway [10]. In January 2011 (halfway in the study period), the catchment area included 398,094 adults 16 years and older. The annual incidence, specified per 10,000 person years, could then be calculated.

The statistical analysis was performed using the Statistical Package for Social Sciences version 22.0 (Armonk, NY: IBM Corp.). Comparison between categorical variables was compared with the *X*^2^ test. *T* tests were used to compare groups with normally distributed continuous variables, while nonparametric variables were analyzed using Mann-Whitney *U* test. Statistical significance was considered for *p* values < 0.05, and all tests were two sided. The data are reported with 95% confidence interval (95% CI) where applicable.

## Results

**Fig. 1:**
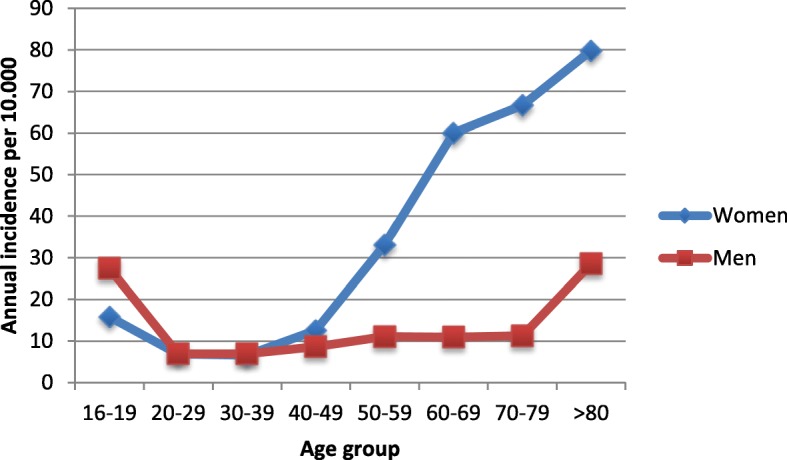
Annual incidence of distal radius fractures per 10,000 persons, 16 years or older

One thousand five hundred sixty-five patients with a distal radius fracture were included in the study. One thousand one hundred thirty-four (72%) of them were women. Median age in men was 47 (16–93) years and 63 (16–98) years in women. Four hundred eighteen (26.7%) fractures were operated. The incidence among women had an exponential growth after the age of 50, with the highest incidence over the age of 80. The incidence in men peaked between the ages 16–19 and above 80 (Fig. 1).

The overall annual fracture incidence was 19.7 (95% CI 18.7–20.7) per 10,000 inhabitants 16 years or older. The incidence was higher among women than men (Table 1).

**Table 1:** Annual incidence of distal radial fractures per 10,000 inhabitants 16 years or older

Gender	Age	Population January 1, 2011	Number of fractures	Annual incidence (95% CI)
Women	16–19	12,908	41	15.9 (9.0–22.8)
	20–29	28,782	40	6.9 (3.9–9.9)
	30–39	36,097	48	6.6 (4.0–9.9)
	40–49	39,223	99	12.6 (9.1–16.1)
	50–59	30,789	204	33.1 (26.7–39.5)
	60–69	27,203	324	60 (50.9–69.1)
	70–79	15,156	202	66.6 (53.7–68.5)
	> 80	11,023	176	79.8 (63.2–96.4)
	Total	201,181	1134	28.2 (25.9–30.5)
Men	16–19	13,639	75	27.5 (18.7–36.3)
	20–29	29,263	41	7.0 (4.0–10.0)
	30–39	35,728	50	7.0 (4.2–9.8)
	40–49	41,261	72	8.7 (5.9–11.5)
	50–59	31,619	70	11.1 (7.4–14.8)
	60–69	26,339	58	11.0 (7.0–15.0)
	70–79	12,780	29	11.3 (5.5–17.1)
	> 80	6284	36	28.6 (15.4–41.8)
	Total	196,913	431	10.9 (9.5–12.3)

Complete data were available for 854 (54.5%) of the 1565 patients where mechanism of injury, fracture classification, and operational method were noted. The most common mechanism of injury was falling while walking outdoors, affecting 447 (52%) patients (Table 2). Ice or snow contributed to 294 (66%) of these injuries. Of all the fractures, walking outdoors contributed to 360 (60%) in women and 87 (34%) in men.

**Table 2:** Mechanism of injury of 854 distal radius fractures treated during 2010 and 2011

Mechanism	Men (%)	Women (%)	Total (%)
Home accidents	24 (9)	106 (18)	130 (15)
Traffic	14 (6)	13(2)	27 (3)
Walking	87 (34)	360 (60)	447 (52)
Accidents at work	40 (16)	6 (1)	46 (5)
Sports	60 (24)	74 (12)	134 (16)
Playing	4 (1)	8 (1)	12 (2)
Other	25 (10)	33 (6)	58 (7)
Total	254 (100)	600 (100)	854 (100)

Women were more often injured indoors while men sustained more injuries at work, in traffic, and during sports.

In our material of 854 wrist fractures, 822 (96.3%) were classified according to the AO system. The remaining 32 fractures were children’s fractures with open growth plates (despite being older than 16 years). These fractures were included in the epidemiological calculations, but could not be classified according to the AO classification system. We found 430 (52%) type A fractures, 97 (12%) type B fractures, and 295 (36%) type C fractures (Table 3).

**Table 3:** Classification of 822 distal radius fractures presenting to Akershus University Hospital during 2010 and 2011

AO type	Women (%)	Men (%)	Total (%)
A	308 (53)	122 (50)	430 (52)
B	76 (13)	21 (9)	97 (12)
C	195 (34)	100 (41)	295 (36)
Total	579 (100)	243 (100)	822 (100)

*AO Arbeitsgemeinschaft fur Osteosyntesefragen*

**Fig. 2:**
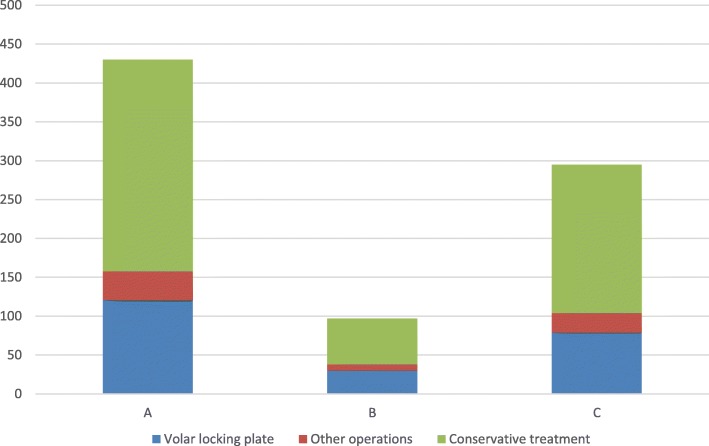
Different treatment methods of distal radius fracture by AO classification

One hundred fifty-nine (37%) of the type A fractures were operated, mostly (121, 76%) with a VLP. One hundred forty-two (36%) of the intra-articular fractures were operated. Among the 97 type B simple intra-articular fractures, 38 (39%) were operated, 30 (79%) of them with a volar locking plate. Finally, of the 295 type C-classified (intra-articular) fractures, 104 (35%) were operated, 79 (76%) with a VLP (Fig. 2).

The difference in number of operations between extra-articular (159 of 430) and intra-articular (142 of 392) fractures was not statistically significant (*p* = 0.8). Seventy-four of the 301 (24.6%) operated patients were primarily considered for conservative treatment.

One hundred seventy-two (80%) of the operated women and 72 (72%) of the operated men were treated with a VLP. This difference was not statistically significant (*p* = 0.1). The rest of the operated patients were treated either with external fixation (EF), with or without additional pins, or by closed reduction and percutaneous pinning (CRPP).

## Discussion

The main finding in this study is the overall incidence of DRFs of 19.7 (95% CI 18.7–20.7) per 10,000 inhabitants 16 years or older. This is lower than what has been seen in other Nordic countries where the incidence has been between 26 [4] and 38 [9] per 10,000 inhabitants.

**Fig. 3:**
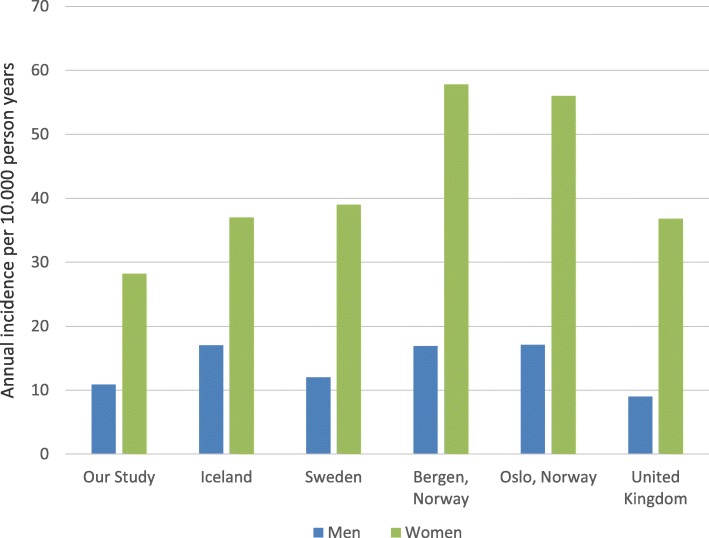
Annual incidence of distal radius fracture among women and men

The incidence among men in our study is relatively close to the findings in both Sweden [4] and the UK [11]. Higher incidences are however described in studies from the Norwegian cities of Bergen [9] and Oslo [2] (Fig. 3).

**Fig. 4:**
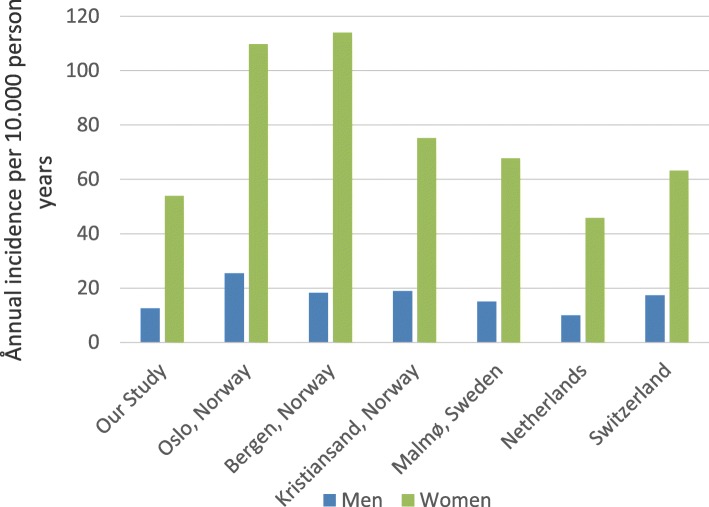
Annual incidence of distal radius fracture among women and men over the age of 50

In women older than 50 years of age, the annual incidence lies relatively close to the reports from the Netherlands [12] and Switzerland [13]. In addition, our findings are just below the reported results from Sweden [4, 14] and the southern part of Norway [1]. Our results are however considerably lower than the Norwegian studies from Oslo and Bergen [2, 9] (Fig. 4).

The studies from Oslo and Bergen were conducted several decades ago. More recent studies show a considerably lower incidence and are closer to our findings [1, 8, 15]. The present study might therefore indicate a true decline in incidence in DRFs. The study population, criteria of inclusion, and study design differ however between the various studies, making comparison challenging. The studies from Bergen and Oslo started their inclusion in patients above the age of 20 years. The incidence of distal radius fractures among children is high, but decreases towards the age of 15–16 years in both genders [16]. In our study, the incidence among women aged 16–19 years is relatively low compared to the other age groups. This will give a low total incidence among women compared to studies not including this group. The incidence in males of the same age group is the second highest, which will increase the total incidence among men.

Elderly are more physically active now than in previous decades, potentially improving both bone mass and neuromuscular control. This could partly explain why our results are lower than previous reports. Geographical differences, as well as snow and ice conditions during the particular winters of our study, may also have reduced the fracture incidence compared to older studies.

We found that the majority (72%) of wrist fractures affect women, which is supported by other studies [1, 2, 4, 8, 9]. As ours and other studies suggest, the distribution was more even among men in the different age groups, with a peak between 16–19 years and above 80 [1, 2, 9] (Fig. 1). Among women, however, we found an exponential increase in incidence between the ages of 50 and 69. It is well established that postmenopausal women have an increased risk of distal radius fractures [1, 2, 4, 8, 9]. This might be explained by reduced bone density and the increased risk of osteoporosis after the menopause [17]. The prevalence of osteoporosis and the risk of fracture is also especially high in the Nordic countries [18, 19] where risk factors such as snow, ice, and slippery roads increase the risk of injury [2, 9, 20].

The exponential growth in our study was followed by a linear increase until the age of 80 (Fig. 1). Similar results have been described in other studies [2, 4, 8, 11]; however, a decrease after the age of 70 has also been suggested [9, 21].

Older people have a tendency to sustain central fractures when falling, such as fractures of the hip and vertebrae, perhaps because of their reduced neuromuscular control. Younger people are more able to protect themselves with their arms and legs and therefore sustain more distal fractures [3, 22]. Recent studies have found the incidence of DRFs to increase also after the age of 70 [2, 4, 8, 11], indicating that older people are living a more active lifestyle, causing fracture of the distal parts of their body more often than before.

The AO type A fracture constituted the majority of the fractures, followed by type C and type B. Our review shows an equal distribution of extra-articular and intra-articular fractures, both genders combined. This tendency was similar to a Dutch report [23]. The results differ from a study from Iceland where 32% of the fractures in patients above the age of 18 involved the articular surface of the distal radius [8]. In Sweden, the results were even lower with 22% [4].

Among males, we found that 50% of the fractures were intra-articular. Similar results are described in the Icelandic study (42%); however, lower results are found in Sweden (29%) [4, 8]. Among Norwegian females, the results were similar, with a small predominance of extra-articular fractures (53%). The prevalence was however lower than the comparing results from Iceland and Sweden, where the extra-articular fractures were more dominating [4, 8]. This difference may be explained by different criteria of inclusion. Both the Swedish and Icelandic studies also included forearm fractures, which will affect the intra- and extra-articular fraction.

Several international studies suggest an increasing tendency for operative treatment [7, 14, 24–26]. Even though most patients were conservatively treated in our study, 418 (26.7%) underwent surgery (Fig. 2). This is a higher percentage than what is found in Sweden (20%) [14] and USA (17%) [24], but comparable to what is previously found in Norway (28%) [25]. We found that the preferred type of surgery was the volar locking plate in 77% of the patients. From 2005 to 2010 in Sweden, the use of VLP tripled and peaked at 67% [14]. A Finnish study found a 108% increased use of VLP over a 10-year period [26]. In Norway, the use of VLP increased from 53% in 2009 to 81% in 2014, while the amount of EF and CRPP went down [25].

Even though the volar locking plate has been increasingly popular and studies have demonstrated good results, there are few randomized controlled trials on this subject. The conclusions are not homogenous regarding what is the optimal surgical treatment [14, 27–33].

The Norwegian Orthopaedic Association published in 2013 guidelines that propose VLP as the preferred implant in most cases. This might explain the increased use in Norway [25].

Our study has some limitations. There is a possibility that not all distal radius fractures have been registered with the code S52.5 (distal radius fracture) or been miscoded. Furthermore, some of the patients belonging to AHUS might have been treated elsewhere. However, AHUS is the only hospital in this area that treats acute injuries and the loss of patients to neighboring hospitals has previously been found to be insignificant [16].

Regarding mechanism of injury, fracture classification, and operational method, there might be a certain selection bias, as the total number of patients with the diagnosis was 1565 and our material consisted of 854 (54.5%) registered patients.

The strengths in our study are the prospective design of the study.

## Conclusions

Compared to earlier Norwegian reports, we found a lower incidence of distal radius fracture. Our numbers are comparable to more recent European studies and might be due to a real decrease in incidence of DRFs.

Postmenopausal women had a higher risk of fractures than the other groups, and low-energy injuries were most dominant. 26.7% of the patients were treated operatively, which is higher than earlier reports, and might reflect an increasing trend for surgical treatment. VLP was the preferred choice of treatment in 77% of the cases.

## Abbreviations

AHUS: Akershus University Hospital
CRPP: Closed reduction and percutaneous pinning
DRF: Distal radius fracture
EF: External fixation
VLP: Volar locking plate

## References

[1] Diamantopoulos AP (2012). The epidemiology of low- and high-energy distal radius fracture in middle-aged and elderly men and women in Southern Norway. PLoS One.

[2] Lofthus C (2008). Epidemiology of distal forearm fractures in Oslo, Norway. Osteoporos Int.

[3] Nellans KW, Kowalski E, Chung KC (2012). The epidemiology of distal radius fractures. Hand Clin.

[4] Brogren E, Petranek M, Atroshi I (2007). Incidence and characteristics of distal radius fractures in a southern Swedish region. BMC Musculoskelet Disord.

[5] Shauver MJ (2011). Current and future national costs to medicare for the treatment of distal radius fracture in the elderly. J Hand Surg.

[6] Melton L (1998). Long-term trends in the incidence of distal forearm fractures. Osteoporos Int.

[7] Wilcke MK, Hammarberg H, Adolphson PY (2013). Epidemiology and changed surgical treatment methods for fractures of the distal radius: a registry analysis of 42,583 patients in Stockholm County, Sweden, 2004–2010. Acta Orthop.

[8] Sigurdardottir K, Halldorsson S, Robertsson J (2011). Epidemiology and treatment of distal radius fractures in Reykjavik, Iceland, in 2004: comparison with an Icelandic study from 1985. Acta Orthop.

[9] Hove LM (1995). Fractures of the distal radius in a Norwegian city. Scand J Plast Reconstr Surg Hand Surg.

[10] Statistisk sentralbyrå. Available from: https://www.ssb.no/en/. Accessed 3 Jan 2018.

[11] Thompson PW, Taylor J, Dawson A (2004). The annual incidence and seasonal variation of fractures of the distal radius in men and women over 25 years in Dorset, UK. Injury.

[12] De Putter C (2013). Epidemiology and health-care utilisation of wrist fractures in older adults in The Netherlands, 1997–2009. Injury.

[13] Lippuner K (2009). Remaining lifetime and absolute 10-year probabilities of osteoporotic fracture in Swiss men and women. Osteoporos Int.

[14] Mellstrand-Navarro C (2014). The operative treatment of fractures of the distal radius is increasing. Bone Joint J.

[15] Hoff M, Torvik IA, Schei B (2016). Forearm fractures in Central Norway, 1999–2012: incidence, time trends, and seasonal variation. Arch Osteoporos.

[16] Randsborg P-H (2013). Fractures in children: epidemiology and activity-specific fracture rates. JBJS.

[17] Cawthon PM (2011). Gender differences in osteoporosis and fractures. Clin Orthop Relat Res.

[18] Gjesdal CG (2004). Femoral and whole-body bone mineral density in middle-aged and older Norwegian men and women: suitability of the reference values. Osteoporos Int.

[19] Kanis JA (2002). International variations in hip fracture probabilities: implications for risk assessment. J Bone Miner Res.

[20] Diamantopoulos AP (2013). Short-and long-term mortality in males and females with fragility hip fracture in Norway. A population-based study. Clin Interv Aging.

[21] Falch J (1983). Epidemiology of fractures of the distal forearm in Oslo, Norway. Acta Orthop Scand.

[22] Vogt MT (2002). Distal radius fractures in older women: a 10-year follow-up study of descriptive characteristics and risk factors. The study of osteoporotic fractures. J Am Geriatr Soc.

[23] Bentohami A (2014). Incidence and characteristics of distal radial fractures in an urban population in The Netherlands. Eur J Trauma Emerg Surg.

[24] Fanuele J (2009). Distal radial fracture treatment: what you get may depend on your age and address. J Bone Joint Surg Am.

[25] Kvernmo HD, Otterdal P, Balteskard L. Treatment of wrist fractures 2009–14. Tidsskr Nor Laegeforen. 2017;137(19):1501–05. 10.4045/tidsskr.17.0065 29043745

[26] Mattila VM (2011). Significant change in the surgical treatment of distal radius fractures: a nationwide study between 1998 and 2008 in Finland. J Trauma Acute Care Surg.

[27] Abramo A (2009). Open reduction and internal fixation compared to closed reduction and external fixation in distal radial fractures: a randomized study of 50 patients. Acta Orthop.

[28] Day CS, Maniwa K, Wu WK (2013). More evidence that volar locked plating for distal radial fractures does not offer a functional advantage over traditional treatment options: commentary on an article by Alexia Karantana, FRCS (Orth), et al. “Surgical treatment of distal radial fractures with a volar locking plate versus conventional percutaneous methods. A randomized controlled trial”. JBJS.

[29] Rozental TD, Blazar PE (2006). Functional outcome and complications after volar plating for dorsally displaced, unstable fractures of the distal radius. J Hand Surg.

[30] Leung F (2008). Comparison of external and percutaneous pin fixation with plate fixation for intra-articular distal radial fractures: a randomized study. JBJS.

[31] Wei DH (2009). Unstable distal radial fractures treated with external fixation, a radial column plate, or a volar plate: a prospective randomized trial. JBJS.

[32] Westphal T (2005). Outcome after surgery of distal radius fractures: no differences between external fixation and ORIF. Arch Orthop Trauma Surg.

[33] Williksen JH (2013). Volar locking plates versus external fixation and adjuvant pin fixation in unstable distal radius fractures: a randomized, controlled study. J Hand Surg.

